# Stiripentol fails to lower plasma oxalate in a dialysis-dependent PH1 patient

**DOI:** 10.1007/s00467-020-04585-5

**Published:** 2020-05-16

**Authors:** Caroline Kempf, Anja Pfau, Johannes Holle, Karen Müller-Schlüter, Philip Bufler, Felix Knauf, Dominik Müller

**Affiliations:** 1 Departments of Pediatric Gastroenterology, Nephrology and Metabolic Diseases, Charité University Medicine, Berlin, Germany; 2 Department of Nephrology and Medical Intensive Care, Charité Universitätsmedizin Berlin, Berlin, Germany; 3 Epilepsy Center for Children, University Hospital Neuruppin, Brandenburg Medical School, Neuruppin, Germany

**Keywords:** Hyperoxaluria, Stiripentol, Metabolic disorders, End-stage renal disease, Dialysis

## Abstract

**Background:**

Primary hyperoxaluria type 1 (PH1) is a multisystemic metabolic disorder caused by an excessive production of oxalate by the liver. The majority of patients presenting in early infancy have end-stage renal disease (ESRD). While awaiting the results of sRNAi trials, the current standard treatment is combined liver-kidney transplantation. Recently, Stiripentol has been reported as a promising drug in the treatment of primary hyperoxaluria by reducing urinary oxalate (U_Ox_). Stiripentol is an anti-convulsive drug used in the treatment of children suffering from Dravet syndrome. It causes blockage of the last step in oxalate production by inhibition of hepatic lactate dehydrogenase 5 (LDH5).

**Case:**

We administered Stiripentol as compassionate use in an anuric infant with dialysis-dependent PH1 over a period of 4 months. Although achieving plasma concentrations of Stiripentol that were recently reported to lower U_Ox_ excretion, we did not observe significant reduction to plasma oxalate concentrations (P_Ox_).

**Conclusion:**

We conclude that Stiripentol may not be useful to reduce P_Ox_ in PH patients with advanced chronic kidney disease (CKD), but larger studies are needed to confirm this finding.

## Background

Primary hyperoxaluria type 1 (PH1) is one of the three genetically classified forms of inherited primary hyperoxalurias [1]. All forms belong to the group of rare metabolic diseases. PH1 is caused by homo- or compound heterozygous mutations in the gene which codes for the hepatic peroxisomal enzyme alanine-glyoxylate aminotransferase (AGXT). As a consequence of a mal- or non-functioning enzyme alanine-glyoxylate aminotransferase (AGT), its substrate, glyoxylate, cannot be converted into glycine but is converted via the enzymes glycolate oxidase (GO) as well as LDH5 into oxalate [1]. The excessive production of oxalate causes deposits particularly in the calcium-oxalate form in the kidney and leads to a mechanical but also inflammatory kidney injury followed by a decrease of glomerular filtration rate (GFR) and progression to ESRD [1, 2]. In the absence of sufficient oxalate excretion by the continuously decreasing kidney function, oxalate accumulates with rising concentrations of plasma oxalate, and crossing the saturation threshold crystalizes throughout the body, e.g., the bone, vessel walls, skin, heart, retina, bone marrow, and central nervous system (systemic oxalosis) [3]. Systemic oxalosis leads to progressive severe illness and death. Vitamin B6 acts as a co-factor of AGT, and therapy with vitamin B6 leads to reduction of endogenous oxalate production in some patients with residual AGT activity. The current standard of PH1 treatment is, except for the vitamin B6-dependent forms, a combined liver and kidney transplantation, either simultaneously or sequentially [4]. As this procedure confers many short- and long-term risks (5-year survival rate is 76%), there is urgent need for alternative, less invasive therapies [5]. Novel approaches of substrate-reduction therapies by RNA interference (RNAi) technology are in development [6]. However, data on the effectiveness of RNAi therapy in patients with advanced kidney disease is not expected until the end of 2020 [7]. Metabolic effects of RNA interference that targets hepatic LDH5 needs further investigation [8].

Stiripentol, a well-known anticonvulsive drug, has been shown to inhibit lactate dehydrogenase 5 isoenzyme (LDH5), targeting glyoxylate transformation. In 2019, Le Dudal and colleagues described (a) the successful use of Stiripentol to lower U_Ox_ excretion in cell cultures and an animal model of oxalate nephropathy, (b) lower U_Ox_ excretion in patients treated with Stiripentol for Dravet syndrome, and (c) decreased U_Ox_ excretion in a PH1 patient with preserved kidney function [9]. With respect to these promising results, we administered Stiripentol in a PH1 patient with ESRD over a period of 4 months and report the results here.

## Case report

**Fig. 1:**
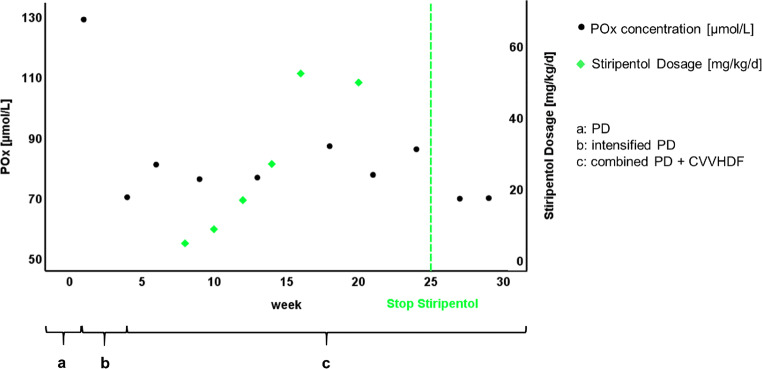
a, PD; b, PD intensified (7 times per week); c, PD and CVVHDF (PD seven times per week, and CVVHDF 5 times per week)

A 17-month-old male boy of northern African origin presented to our hospital. At that time, he was in established renal failure, having been commenced on peritoneal dialysis (PD) at the age of 9 months in his home country. Diagnosis of PH1 has been confirmed within the first year of life by genetic analysis revealing a homozygous mutation in the AGXT gene (Ile244Thr). We measured free plasma oxalate (P_Ox_) concentrations following a protocol previously established in our laboratory. Using this protocol, P_Ox_ concentrations between 20 and 40 μmol/L are commonly obtained in general ESRD patients [10]. In our patient, P_Ox_ concentration was 128.9 μmol/L at presentation. In the further course, samples for control of P_Ox_ concentration were taken after nocturnal peritoneal dialysis and pre-hemodialysis. The patient demonstrated slight pancytopenia, but no signs of retinopathy, echocardiography, and Speckle tracking echocardiography were unremarkable. He was initially anuric, and during the course of his hospitalization, urine output slightly improved (less than 0.1 ml/kg BW/h). The patient had already received pyridoxine (9 mg/kg BW per day) for several months prior to admission as responsiveness to this medication has been described in patients with the Ile244Thr genotype [1]. As his PD program has been insufficient to effectively lower P_Ox_ concentration, we first introduced a daily program (10 dwells of 80 min with 300 mL each, Glc 1.5%; Ca^2+^ 1.25%) according to GPN standard. In addition to PD, we placed a tunneled catheter via the right vena jugularis interna and started our patient on continuous venovenous hemodiafiltration (CVVHDF, 5 sessions of 2–3 h per week; dialysate flow 5-7 L/h; blood flow 50–60 mL/min). Following initiation of this regimen, P_Ox_ concentration dropped to 80.8 μmol/L (Fig. 1). For the compassionate use of Stiripentol, the parents gave written informed consent. Subsequently, Stiripentol was introduced with a starting dosage of 4.7 mg/kg/d and increased stepwise up to 61 mg/kg/d for the duration of 2 months. At the end of the trial, we administered a dosage slightly above the recommended dosage; however, Stiripentol plasma measurements by high-performance liquid chromatography showed values within a non-toxic range. The manufacturer’s recommendations were followed specifically concerning dietary precautions. Most frequent side effects (≥ 1/100) of Stiripentol are loss of appetite and weight, dizziness, insomnia, irritability, hyperkinesia, nausea and vomiting, elevated GGT, and neutropenia. Adverse reactions were not observed in our patient. During the whole 3.5-month period of Stiripentol administration, we did not observe a decrease in P_Ox_ concentration and, therefore, stopped administration. Four weeks after discontinuation, P_Ox_ concentrations were found to remain within the same range, suggesting that the withdrawal did not increase oxalate load further.

## Conclusion

PH1 is a rare life-threatening disorder. With the exception of responsiveness to pyridoxine, mostly in patients with the Gly170Arg and Phe152Ile mutations, and its mediated effects like protein stability, catalytic activity, and peroxisomal transport, there is no drug known to improve or even to stabilize this condition. Also, association between age at onset and mutation is of high intrafamilial phenotypic heterogeneity [3].

Stiripentol has recently been reported as being effective in a PH1 cell culture model and in patients suffering from Dravet syndrome and PH1 [9]. Despite administering a dosage that is comparable with that administered to Dravet patients and the patient reported by Le Dudal et al., we were not able to demonstrate lowering of P_Ox_ concentrations. In contrast to the patient reported in the literature, our patient suffered from more advanced disease when Stiripentol administration was initiated. Stiripentol dosage and plasma concentrations were within the reference ranges [11]. Therefore, there are different considerations to explain our findings: the effect of Stiripentol on the reduction of P_Ox_ concentration might be less powerful than expected and might only be detectable in patients with mild courses of PH1. Alternatively, the inhibition of LDH 5 by Stiripentol might be depending on eGFR, so only patients with preserved kidney function in the early course of disease might benefit from its therapy.

Our findings provide evidence that Stiripentol is not useful in our patient; however, larger studies have been initiated to demonstrate the efficacy of Stiripentol in patients suffering from PH [12]. In contrast to options of GO inhibiting therapy for PH1 patients, blockage of the last step of oxalate production by inhibition of LDH5 could also be of therapeutic value for patients with PH2 or PH3. It is necessary to establish if Stiripentol is capable of lowering P_Ox_ concentrations consistently in PH patients and if this relates to the level of renal dysfunction. Moreover, Stiripentol may need to be compared with other RNA interference substrate-reduction therapies to demonstrate whether it achieves sufficient inhibition of LDH5 to substantially reduce P_Ox_ generation in PH1 patients in the different stages of CKD.
